# Supplementary material for: In Silico/In Vitro Hit-to-Lead Methodology Yields SMYD3 Inhibitor That Eliminates Unrestrained Proliferation of Breast Carcinoma Cells
Source: Int J Mol Sci. 2020 Dec 15;21(24):9549. doi: 10.3390/ijms21249549 (PMC7765450; doi:10.3390/ijms21249549)
Supplement: Supplementary file 1 [file ijms-21-09549-s001.pdf]

In Silico/In Vitro Hit-to-Lead Methodology Yields SMYD3 Inhibitor That Eliminates Unrestrained Proliferation of Breast Carcinoma Cells

Ilham M. Alshiraihi [1,2], Dillon K. Jarrell [3], Zeyad Arhouma [1,4], Kelly N. Hassell [1], Jaelyn Montgomery [5], Alyssa Padilla [5], Hend M. Ibrahim [6,7,8], Debbie C. Crans [1,4], Takamitsu A. Kato [1,7] and Mark A. Brown [1,8,9,10,11,*]

1 Cell and Molecular Biology Program, Colorado State University, Fort Collins, CO 80523-1005, USA; alshiraihi@gmail.com (I.M.A.); zkrahuma@rams.colostate.edu (Z.A.); khassell@colostate.edu (K.N.H.); debbie.crans@colostate.edu (D.C.C.); tkato@rams.colostate.edu (T.A.K.)
2 Department of Biology, University of Tabuk, Tabuk, Tabuk 47713, Saudi Arabia
3 Department of Bioengineering, University of Colorado Anschutz Medical Campus, Aurora, CO 80045-7109, USA; dillom.jarrel@cuanschutz.edu
4 Department of Chemistry, Colorado State University, Fort Collins, CO 80523-1872, USA
5 Department of Biomedical Sciences, Colorado State University, Fort Collins, CO 80523-1617, USA; aepad20@rams.colostate.edu
6 Department of Medical Biochemistry, Zagazig University, Zagazig 44511, Egypt; hendibrahim1@gmail.com
7 Department of Environmental & Radiological Health Sciences, Colorado State University, Fort Collins, CO 80523-1618, USA
8 Department of Clinical Sciences, Colorado State University, Fort Collins, CO 80523-1678, USA
9 Epidemiology Section, Colorado School of Public Health, Fort Collins, CO 80523-1612, USA
10 Institute for Learning and Teaching, Colorado State University, Fort Collins, CO 80523-1052, USA
11 Department of Ethnic Studies, Colorado State University, Fort Collins, CO 80523-1790, USA
* Correspondence: mark.brown@colostate.edu

(a)

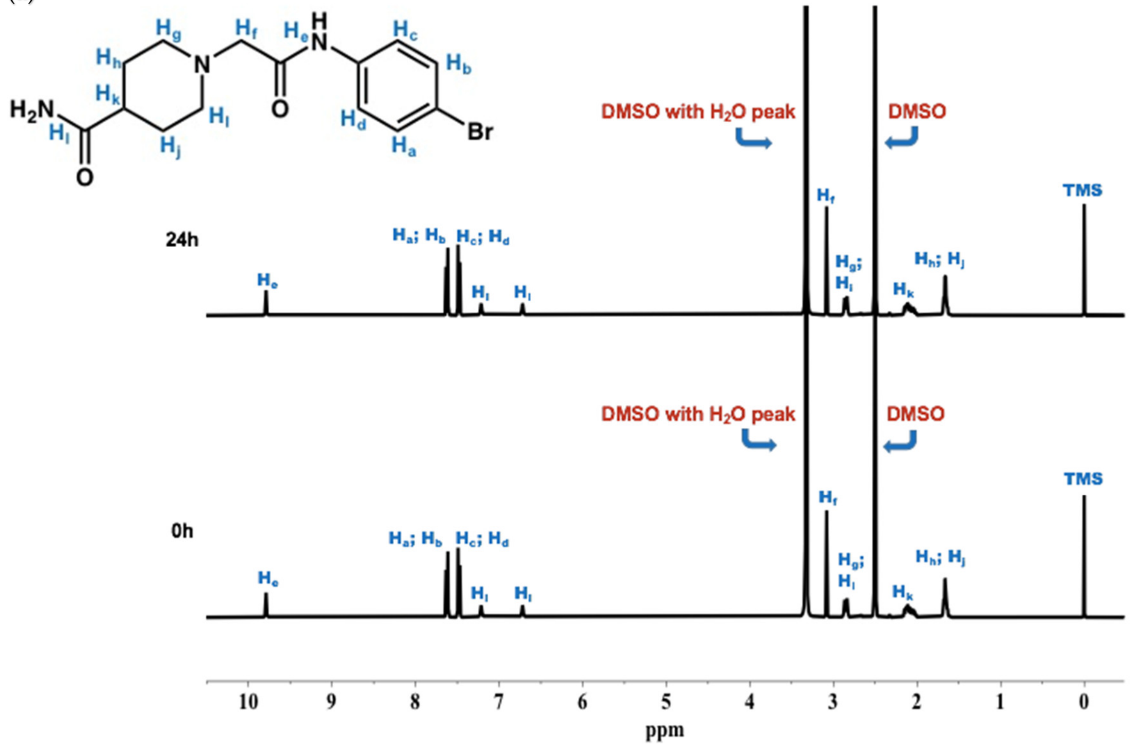

(b)

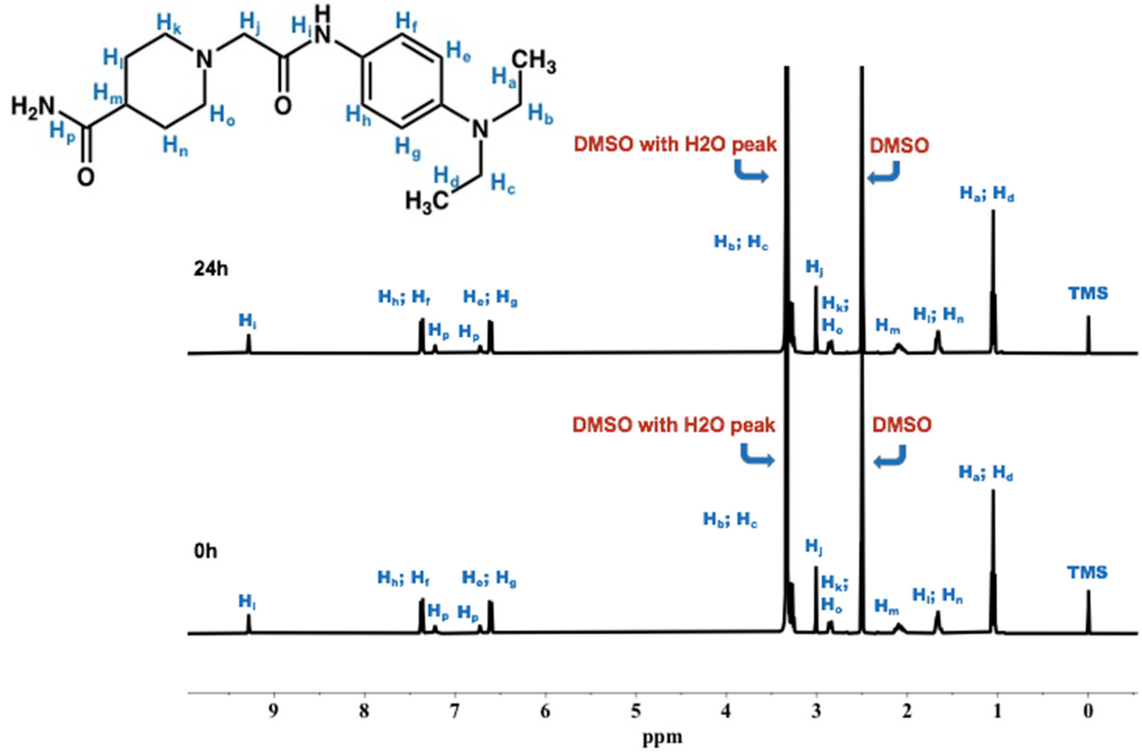

S-Figure 1. ^{1}H NMR spectra of fresh (0h) and aged (24h) of **(a)** BCI-121 and **(b)** inhibitor-4 in d_6-DMSO

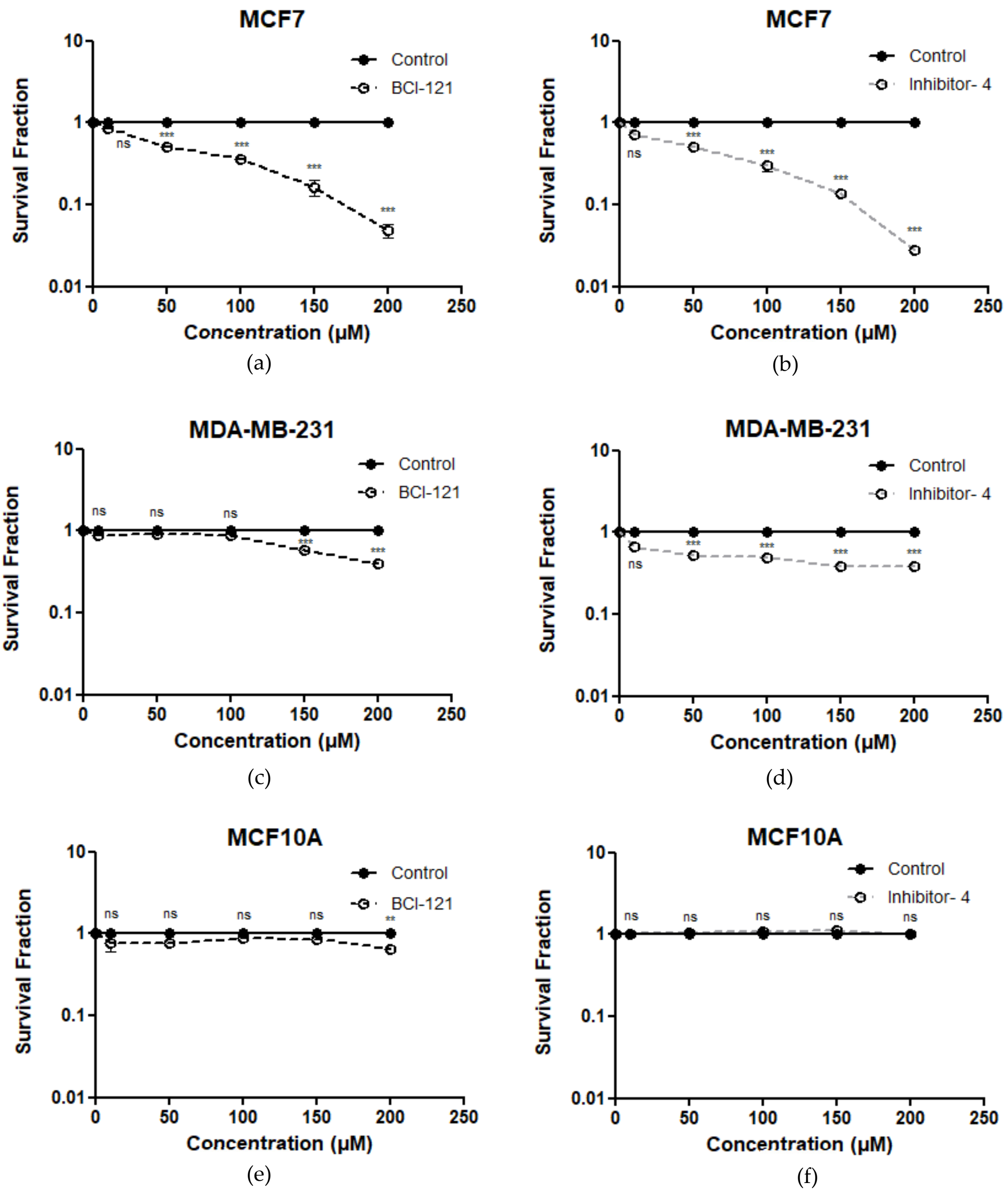

S-Figure 2. Clonogenic cell survival curve against BCI-121 (positive control inhibitor) and Inhibitor-4. (a, c, e) BCI-121 impact on breast cancer cell lines (a and c) and normal breast epithelial cell line (e). (b, d, f) Inhibitor-4 impact on breast cancer cell lines (b and d) and normal breast epithelial cell line (f). Error bars display standard error of means. Statistically significant differences from control are indicated by ** $P < 0.01$, *** $P < 0.001$ *or* ns $P > 0.05$. At least three independent experiments were carried out.

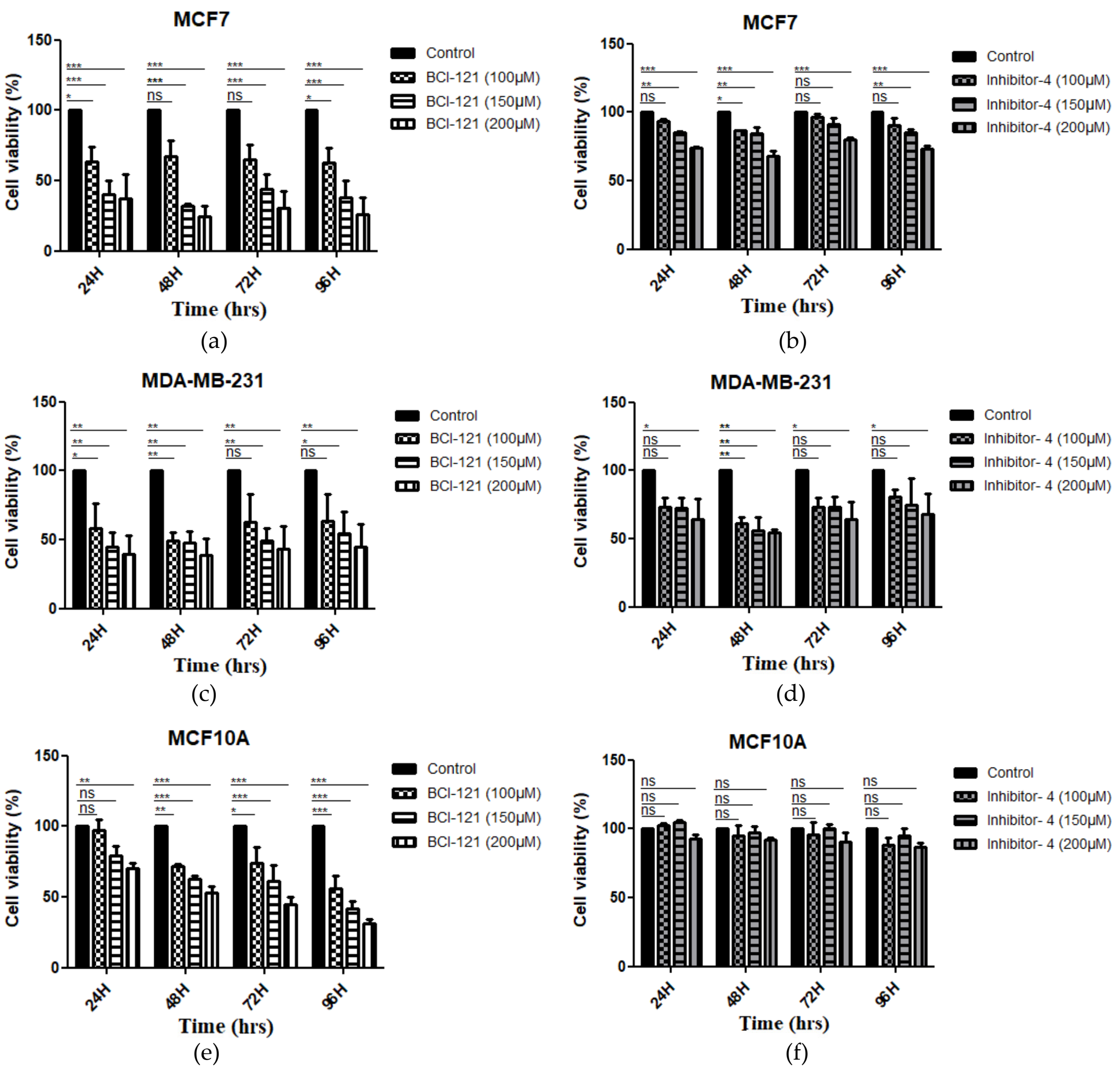

S-Figure 3. Cell viability using MTT assay. (a, c and e) BCI-121 effect on MCF7, MDA-MB-231 (breast cancer cell lines) and MCF10A (normal breast epithelial cell line). (b, d and f) Inhibitor-4 impact on MCF7, MDA-MB-231 (breast cancer cell lines) and MCF10A (normal breast epithelial cell line). Values are mean ± standard error of the means. Statistically significant differences from control are indicated by * *P < 0.05*, ** *P < 0.01*, *** *P < 0.001 or* ns *P > 0.05*.

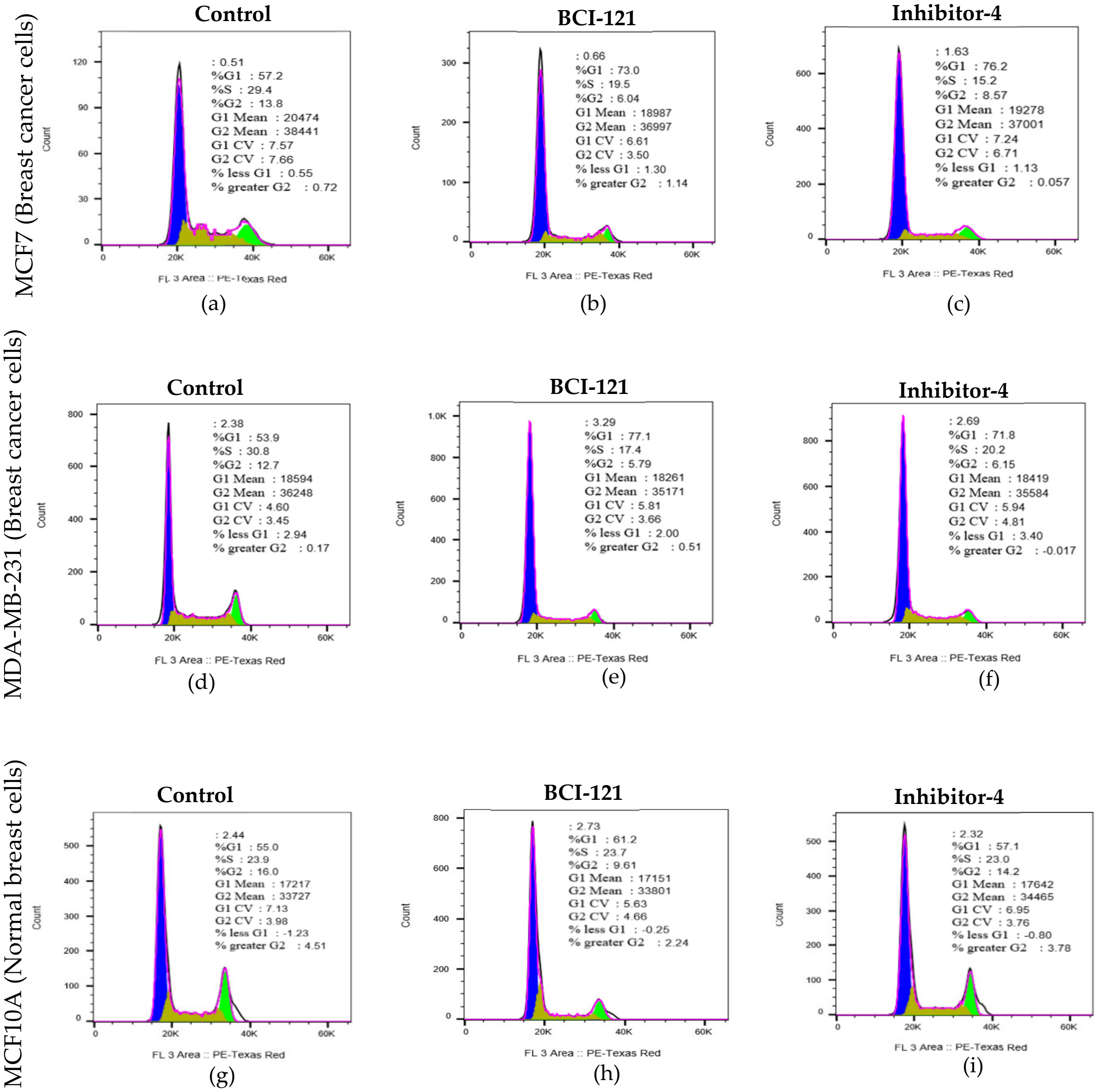

S-Figure 4. The cell cycle distribution was assessed using PI in MCF7, MDA-MB-231 (breast cancer cell lines) and MCF10A cells (normal breast epithelial cell line) with SMYD3 inhibitor treatments for 24 hours and was investigated by flow cytometry. (a, d and g) Untreated control distributions for MCF7, MDA-MB-231 and MCF10A. (b, e and h) Impact of BCI-121 on MCF7, MDA-MB-231 and MCF10A. (c, f and i) Effect of Inhibitor-4 on MCF7, MDA-MB-231 and MCF10A.

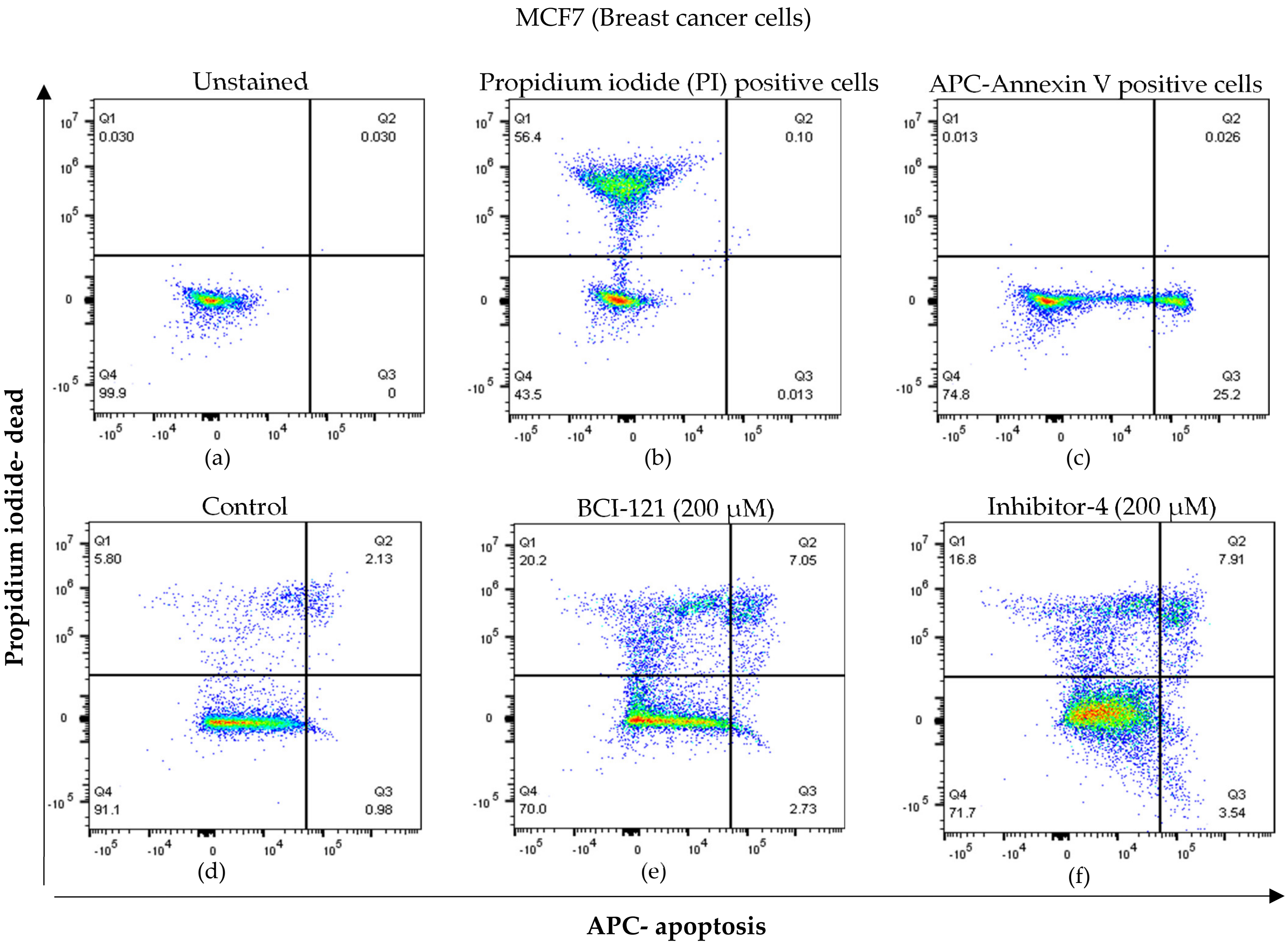
MCF7 (Breast cancer cells)
Unstained
Propidium iodide (PI) positive cells
APC-Annexin V positive cells
Control
BCI-121 (200 µM)
Inhibitor-4 (200 µM)
Q1
Q2
Q3
Q4
0.030
0.030
99.9
0
56.4
0.10
43.5
0.013
0.013
0.026
74.8
25.2
5.80
2.13
91.1
0.98
20.2
7.05
70.0
2.73
16.8
7.91
71.7
3.54
(a)
(b)
(c)
(d)
(e)
(f)
Propidium iodide- dead
APC- apoptosis

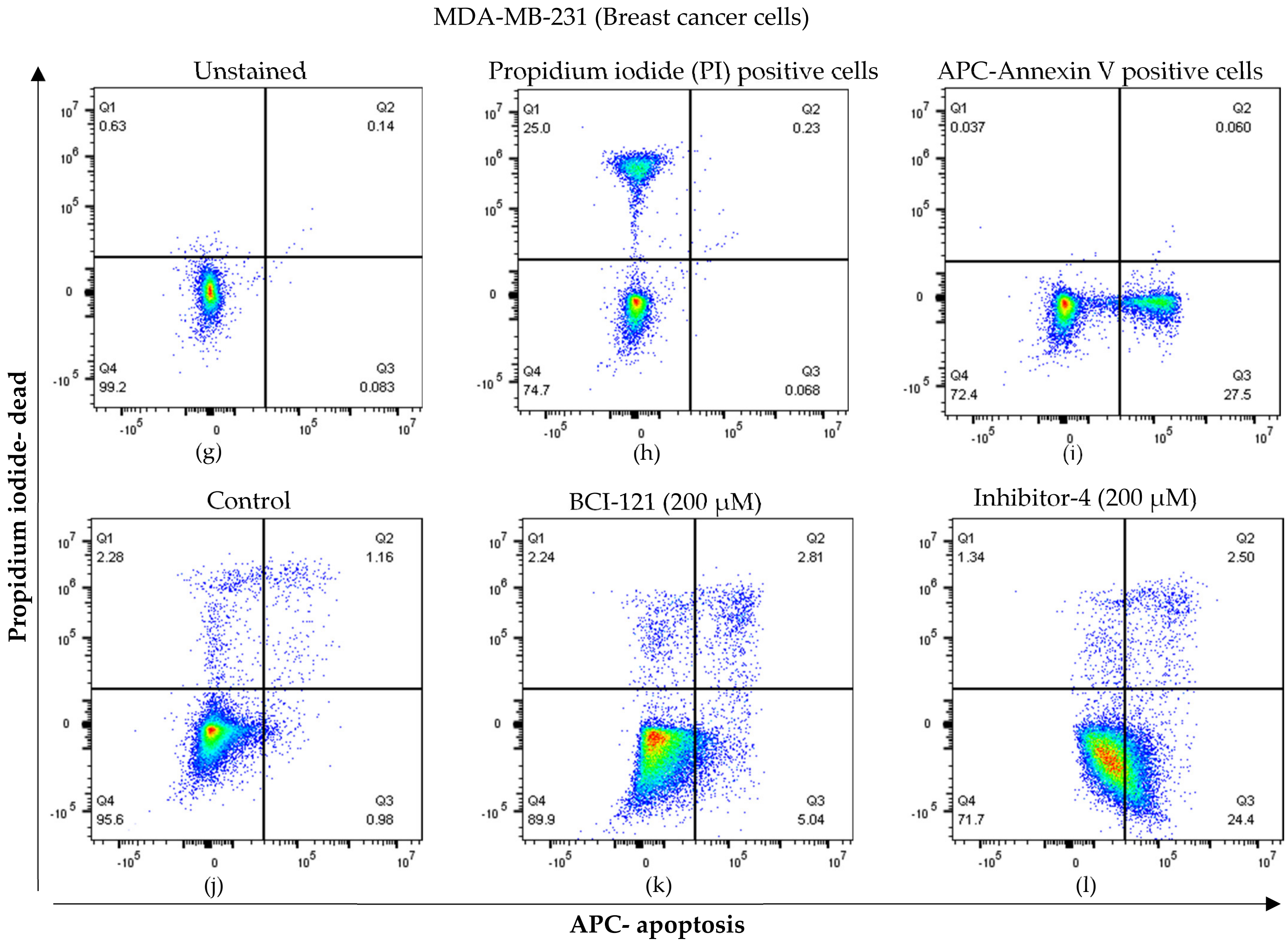

MDA-MB-231 (Breast cancer cells)
Unstained
Propidium iodide (PI) positive cells
APC-Annexin V positive cells
Q1 0.63
Q2 0.14
Q4 99.2
Q3 0.083
(g)
Q1 25.0
Q2 0.23
Q4 74.7
Q3 0.068
(h)
Q1 0.037
Q2 0.060
Q4 72.4
Q3 27.5
(i)
Control
BCI-121 (200 μM)
Inhibitor-4 (200 μM)
Q1 2.28
Q2 1.16
Q4 95.6
Q3 0.98
(j)
Q1 2.24
Q2 2.81
Q4 89.9
Q3 5.04
(k)
Q1 1.34
Q2 2.50
Q4 71.7
Q3 24.4
(l)
Propidium iodide- dead
APC- apoptosis

MCF10A (Normal breast cells)

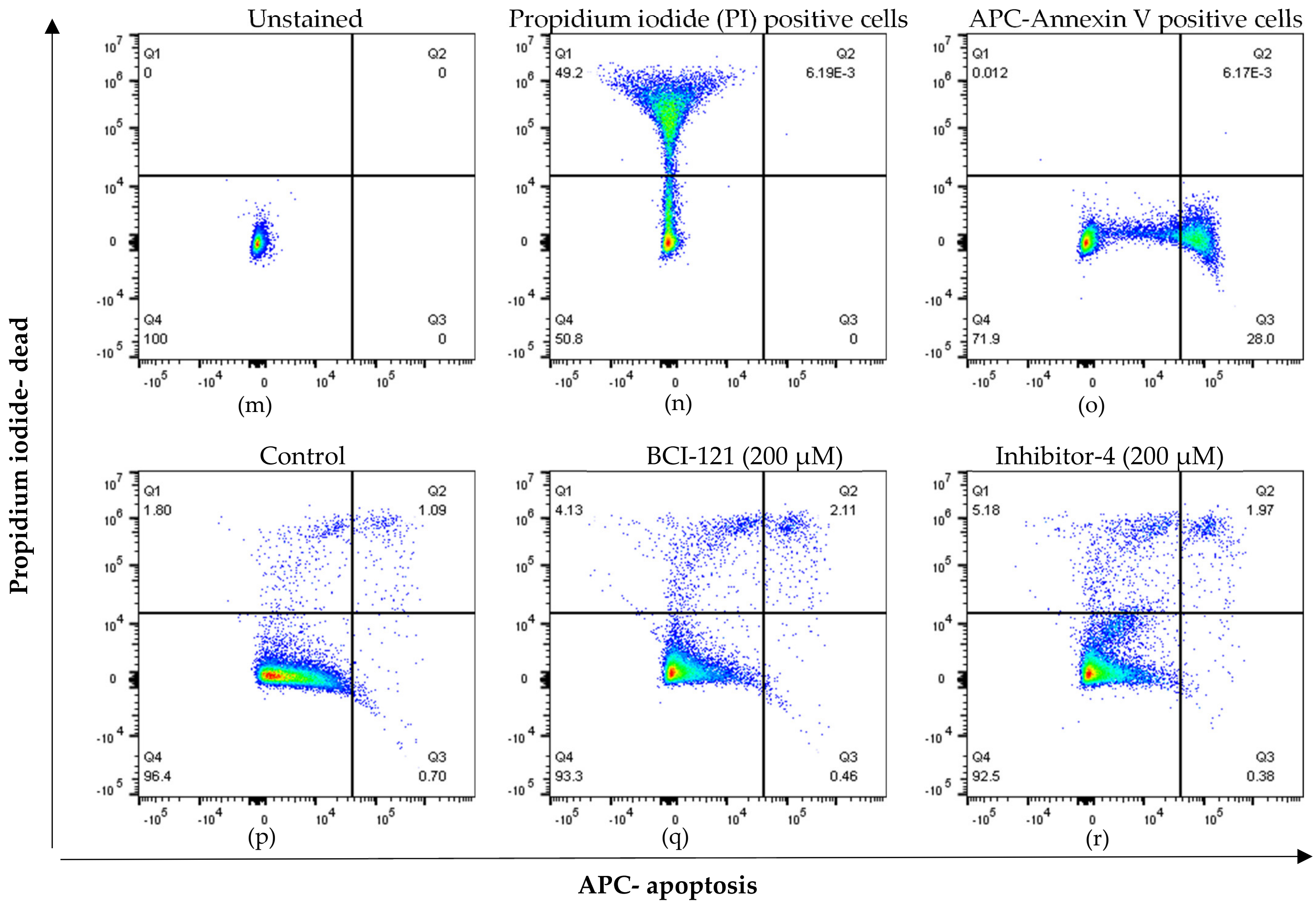

S-Figure 5. Cell apoptosis was assessed using APC Annexin V/PI and flow cytometry. MCF7 (a-f, breast cancer), MDA-MB-231 (g-l, breast cancer) and MCF10A (m-r, normal breast) cell lines were treated with SMYD3 inhibitors for 48 h. (a, g and m) Unstained cells from each line. (b, h and n) PI-positive cells from each line. (c, i and o) APC Annexin V-positive cells. (d, j and p) Control (untreated) cells after 48 hours. (e, k and q) Apoptosis after 48 h BCI-121 treatment on each cell line. (f, l and r) Apoptosis after 48 hours Inhibitor-4 treatment on each cell line.
